# NADPH oxidase 4 and its role in the cardiovascular system

**DOI:** 10.1530/VB-19-0014

**Published:** 2019-07-11

**Authors:** Stephen P Gray, Ajay M Shah, Ioannis Smyrnias

**Affiliations:** 1 School of Cardiovascular Medicine & Sciences, King’s College London British Heart Foundation Centre, London, UK

**Keywords:** NOX4, heart, ROS, vasculature

## Abstract

The heart relies on complex mechanisms that provide adequate myocardial oxygen supply in order to maintain its contractile function. At the cellular level, oxygen undergoes one electron reduction to superoxide through the action of different types of oxidases (e.g. xanthine oxidases, uncoupled nitric oxide synthases, NADPH oxidases or NOX). Locally generated oxygen-derived reactive species (ROS) are involved in various signaling pathways including cardiac adaptation to different types of physiological and pathophysiological stresses (e.g. hypoxia or overload). The specific effects of ROS and their regulation by oxidases are dependent on the amount of ROS generated and their specific subcellular localization. The NOX family of NADPH oxidases is a main source of ROS in the heart. Seven distinct Nox isoforms (NOX1–NOX5 and DUOX1 and 2) have been identified, of which NOX1, 2, 4 and 5 have been characterized in the cardiovascular system. For the purposes of this review, we will focus on the effects of NADPH oxidase 4 (NOX4) in the heart.

## NOX4 variants, activity and localization

NOX4 is a dual heme-containing transmembrane oxidoreductase that spans the membrane six times. NOX4 exists as a heterodimer bound to a p22^phox^ subunit, which is necessary for its activity (1). In contrast to other NOX isoforms, NOX4 does not require any cytosolic regulatory subunit for its activity and is constitutively active with its regulation being a direct consequence of its abundance and intracellular localization (Table 1 for activity, regulation and expression of the main NOXs in the cardiovascular system). Under physiological conditions, NOX4 was first identified and has its highest levels of expression in kidney proximal tubular cells (2), but is also expressed in many other cell types, including cardiomyocytes, endothelial and smooth muscle cells, osteoclasts, epithelial cells and hemopoietic stem cells; albeit at lower levels. Interestingly, NOX4 is encoded by a gene which contains 34 introns and is transcribed into 16 spliced variants, of which at least four generate proteins (NOX4B–E) (3). In particular, NOX4D is the only variant that has been found to be functionally active in terms of ROS generation, despite lacking putative transmembrane regions as it retains the NADPH- and FAD-binding domains required for electron transfer activity. Hence, NOX4D can modulate redox-sensitive transcriptional regulation downstream of ERK1/2 phosphorylation and induces nuclear DNA damage (4). However, further studies are required to delineate the pathophysiological effects of these NOX4 variants. Adding to NOX4 variation, using the standard human *NOX4* gene sequence for comparison, there have been more than 2300 SNP sites found in the genomic DNA region of *NOX4*, and 45 SNPs in the gene-coding region. These SNPs may affect gene replication, transcription and even NOX4 function that may determine the progress and/or development of disease. For instance, polymorphism of rs1836882 in the *NOX4* gene modulates associations between dietary caloric intake and ROS levels in peripheral blood mononuclear cells (5). In the cardiovascular system, the *NOX4* rs11018628 polymorphism has been associated with a decreased risk and better short-term recovery of ischemic stroke (6). More studies are needed to better understand connections between polymorphisms of *NOX4* in different populations and disease-related NOX4 variants.

**Table 1:** The main NOXs in the cardiovascular system.

	Activity	Regulatory subunits/requirement for p22^phox^	Regulation by	Cell expression
NOX1	Inducible	NOXO1, NOXA1, Rac/yes	Post-translational modification of regulatory subunits	Vascular smooth muscle, endothelial cells
NOX2	Inducible	P47^phox^, p67^phox^, p40^phox^, Rac/yes	Post-translational modification of regulatory subunits	Cardiomyocytes, endothelial cells, fibroblasts, vascular smooth muscle cells, inflammatory cells
NOX4	Constitutively active	None/yes	Poldip2 and transcriptional regulation	Cardiomyocytes, endothelial cells, fibroblasts, vascular smooth muscle cells
NOX5	Low constitutive activity	None/no	Ca^2+^	Vascular smooth muscle and endothelial cells (absent in rodents)

In the cardiovascular system, several conditions, such as pressure overload, hypoxia and inflammation lead to increased NOX4 expression, significantly impacting cellular function. Adding to its distinct characteristics over other NOXs, NOX4 primarily produces hydrogen peroxide rather than superoxide due to the presence of an E-loop in its structure that promotes the rapid dismutation of superoxide before it leaves the enzyme (7). In addition to the type of ROS generated by NOX4, its subcellular localization also influences various NOX4 functions, including enzyme activity and the activation of distinct downstream signaling pathways (8, 9). However, the exact location of NOX4 remains largely debated, with reports positioning the enzyme in the endoplasmic reticulum, mitochondria, plasma membrane and nucleus (10, 11). The reasons for these disparities may reflect the cell-specific differences in the functions of NOX4 in the different cell types studied, the fact that NOX4 localization might be transitory based on its interactions with certain targets (12) and/or the quality of research tools and approaches employed.

## NOX4 in the stressed heart

The role of NOX4 in the heart has been characterized in various cardiac disease models with the use of systemic and/or cardiomyocyte-specific NOX4 overexpression or deletion animal models. A summary of the literature is included in Table 2. Several studies report a protective role of NOX4 in models of cardiac hypertrophy and against cardiac remodeling under conditions of stress. The functional benefits of increased NOX4 levels in the pressure-overloaded heart were first identified by Zhang *et al*. when they employed loss- and gain-of-function NOX4 mouse models and reported that, following abdominal aortic banding in mice, NOX4 exerts its protective effects through a mechanism involving paracrine enhancement of capillary density (13). Contrasting observations were reported by the Sadoshima laboratory when they reported the detrimental effects of NOX4 in the overloaded heart due to increased mitochondrial ROS production and damage (14). While these differences may be attributed to the type and severity of overload studied and means via which NOX4 levels were manipulated, the protective effects of NOX4 have been since corroborated in cardiomyocyte- and endothelial-specific NOX4-null mice, where trans-aortic constriction was associated with more severe cardiac function and remodeling in the NOX4-deficient mice (15). Further adding to the protective roles of NOX4 in cardiomyocytes under stress, studies have described the reliance of NOX4 on the antioxidant transcription factor nuclear factor erythroid 2-related factor 2 (NRF2) (16, 17), as well as the NOX4-derived ROS production in the ER and subsequent activation of autophagy, which ensures cell survival during energy deprivation (18).

**Table 2:** NOX4 in cardiac disease models.

NOX4 modification (cardiac disease models)	Disease model	Reported outcome	Reference
Cardiomyocyte-specific overexpression	Pressure overload	Reduced fibrosis and levels of hypertrophy	(13)
Global deletion	Pressure overload	Contractile dysfunction, severe dilatation, increased levels of hypertrophy	(13)
Cardiomyocyte-specific deletion	Pressure overload	Reduced levels of hypertrophy, fibrosis and cell death	(14)
Cardiomyocyte-specific deletion	Pressure overload	Increased levels of hypertrophy and fibrosis, diminished angiogenesis, contractile dysfunction	(15)
Endothelial-specific deletion	Pressure overload	Increased levels of hypertrophy and fibrosis, contractile dysfunction	(15)
Cardiomyocyte-specific overexpression	Pressure overload	Reprogramming of cardiac metabolism to fully maintain energetic status	(63)
Global deletion	Ischemia/reperfusion	No NOX4-dependent effects	(19)
Global deletion	Ischemia/reperfusion	Severe cardiac lesions	(21)
Cardiomyocyte-specific overexpression	Permanent left anterior descending ligation	Improved contractile function, reduced cardiac remodeling	(64)
Cardiomyocyte-specific deletion	Ischemia/reperfusion	Decreased myocardial damage, reduced ROS production, attenuation of infarct size	(20)

Whereas the protective role of NOX4 in the chronically overloaded heart is well established, contrasting results have been reported on the role of NOX4 in ischemia/reperfusion (IR) injury. Braunersreuther *et al*. have reported that NOX4 deletion does not influence myocardial reperfusion injury while demonstrating the activation of cardioprotective pathways following ablation of NOX1 and NOX2 (19). In another study, Matsusima *et al*. demonstrated a decrease in myocardial damage following IR in cardiac-specific NOX4-deficient mice, which was associated with reduced ROS production and an attenuation of the infarct size, suggesting that NOX4 actually mediates IR injury (20). However, myocardial injury was exacerbated in the NOK2-/NOX4-deficient mice, suggesting that a certain amount of ROS produced by either NOX2 or NOX4 is necessary for protection against IR injury. Moreover, a study by Santos *et al*. shows extensive data on a NOX4-regulated pathway involving inactivation of the protein phosphatase 1 (PP1) and sustained eIF2α phosphorylation, which regulates the transcription factor ATF4 and enhances cell survival in heart IR injury. This novel redox signaling pathway involves an interaction between NOX4, growth arrest and DNA damage-inducible 34 (GADD34) to inactivate the protein phosphatase 1 (PP1) metal center and sustain eIF2α phosphorylation, eventually protecting the heart under stress (21). Further studies are required to delineate some of these discrepancies on the exact role of NOX4 during IR injury in the heart.

## NOX4 and the vasculature

A summary of the literature describing the role of NOX4 in vascular disease models is included in Table 3. Most pathologies of the vasculature start with endothelial dysfunction (ED) increasing the likelihood of developing hypertension (22, 23). NOX4 has been demonstrated to be an important vasodilator and can act as an endothelium-derived hyperpolarizing factor (24, 25). H_2_O_2_ has been shown to increase endothelial NOS expression and activity (26), enhancing NO production (27). A role for NOX4 in hypertension is contentious and has not yet been conclusively determined (28, 29). Endothelial cell (EC)-specific overexpression of NOX4 enhanced agonist-mediated vasodilatation resulting in a decrease in basal blood pressure (BP) (30). This effect was mediated through the vasodilatory actions of H_2_O_2_ and not by increased NO bioavailability (31). In agreement, Paravicini *et al*. (32) showed that NOX4 expression in basilar arteries was associated with enhanced vasodilatation in response to H_2_O_2_-mediated activation of BK(Ca) channels. Conversely, a number of studies have reported no change in BP (33, 34, 35, 36). Such is the recent study by Bouabout *et al.* (37), which demonstrated no change in BP at baseline in NOX4-deficient mice, but a protection in Ang-II mediated arterial and pulse pressure increases. Taken together, these findings suggest that while NOX4 has been demonstrated to be involved in the regulation of hypertension, its effects could be cell and disease specific.

**Table 3:** NOX4 in vascular disease models.

NOX4 modification (vascular disease models)	Disease model	Reported outcome	Reference
Overexpression	Endothelial Dysfunction	Enhanced agonist-mediated relaxation eNOS-dependent acceleration in neovascularization in hind limb ischemia	(30, 38)
Global deletion	Hypertension	No change in BP at baseline but a protection in Ang-II mediated pressure increases	(37)
Global deletion	Endothelial dysfunction	Reduced contractile dysfunction	(14)
Global deletion	Atherosclerosis	Accelerated development in diabetic model	(34, 35)
Global deletion	Ischemia/reperfusion and Stroke	Reduction in ROS and less blood–brain barrier leakage	(39)
Global deletion	Atherosclerosis	Reduced development of the neointima	(14)

Atherosclerosis development involves multiple cell types, which all express NOX4 at basal levels and as such it is expected that NOX4 plays a role; albeit several studies have suggested both an athero-protective (30, 38, 39, 40) and a deleterious role (41, 42, 43, 44, 45). The induction of growth factors and cytokines in the vessel have been shown to be regulated by NOX4 (40, 46, 47) and that NOX4 has been implicated in neointima formation after vascular injury. Specifically, knockdown of NOX4 in Zucker rats reduced SERCA oxidation and inhibited the development of the neointima in carotid injury (14). Moreover, oxidized LDL stimulates NOX4 expression in macrophages, a process that leads to necrotic core formation within lesions (48). Furthermore, NOX4 has been linked to smooth muscle cell (SMC) migration and proliferation, which are essential steps in the development of atherosclerosis (42, 49). Xu *et al.* (43) reported that NOX4 expression was increased in aged atherosclerotic plaques, specifically in the SMC of unstable plaques, through an increase in SMC senescence and apoptosis (43), an important step in the development of unstable lesions. It has also been demonstrated that in the setting of diabetes, NOX4 deletion results in a dedifferentiation of the SMC and increased proliferation (49). Additionally, STZ-diabetic NOX4-/ApoE-deficient mice have no change in atherosclerosis development after 10 weeks (34); however, after 20 weeks of diabetes, there was a significant elevation in atherosclerotic development through increased SMC proliferation (35). Furthermore, EC-specific overexpression of the human NOX4 dominant negative P437H mutant led to an acceleration in atherosclerosis development and a cell-specific decline in NOX4 expression in the EC vs SMC of STZ-diabetic mouse vessels (50). These findings indicate that NOX4 in the setting of atherosclerosis appears to work in a time-/cell-/disease-specific manner and that overall NOX4 appears to play an athero-protective role.

Transient or sustained ischemia can lead to infarcts and stroke within the cerebral vasculature. Similar to the reports in the pressure-overloaded heart, NOX4 has been linked to the pathophysiology of stroke, since its expression and activity is increased as a consequence of hypoxia (51, 52). NOX4 is upregulated in the cortical neurons within 24 h of middle cerebral artery occlusion (51). Transient upregulation of NOX4 in the cortex is also observed after endothelin-induced stroke (53). In an extensive study conducted by Kleinschnitz *et al.* (39), NOX4-deficient mice had less oxidative stress, less blood–brain barrier leakage and less neuronal apoptosis after either transient occlusion of the middle cerebral artery or after permanent stroke induced by cortical photothrombosis. Importantly, post-stroke treatment with the putative NOX inhibitor VAS2870 improved recovery, suggesting that NOX4 may be a viable therapeutic target in the setting of stroke (39). This notion has gained further support in a recent study, which identified an increase in infarct size after middle cerebral artery occlusion in addition to a reduction in endothelial-derived eNOS when NOX4 oxidase was overexpressed in EC (54). The contrasting findings in the setting of stroke compared to the setting of atherosclerosis highlight that NOX4 can play both a detrimental and protective role in disease development and that this may largely depend on the specific nature of the vessel, that being macrovascular or microvascular. This highlights the need for further research into the role of NOX4 in other vascular beds, before using blanket NOX4 inhibitors to modulate disease development.

## NOX4-mediated regulation of transcription factors in the heart

Several studies have reported the ability of NOX4 to regulate distinct signaling pathways and cellular functions (e.g., proliferation (55), apoptosis (56), angiogenesis (13) and more) based on its levels of expression, intracellular localization and the cell type studied. For instance, among others NOX4 has been shown to activate the kinases p38, JNK, ERK1/2 and Akt in both stimulated and naïve cells (57, 58, 59). In the cardiovascular system, NOX4 has been shown to convey several of its actions via interaction with different transcription factors such as NRF2, HIF1a and ATF4. NRF2 is a pleiotropic transcription factor primarily acting as a central regulator of an antioxidant cytoprotective gene program that can be activated in cardiomyocytes during acute neurohumoural stress or in the overloaded heart *in vivo*. Overexpression of NOX4 *in vivo* has been shown to mediate the expression of antioxidant and detoxifying genes regulated by NRF2, as well as an NRF2-dependent elevation of glutathione and biosynthetic and recycling enzymes, suggesting a role for NOX4 in the regulation of glutathione redox in the heart (16). Furthermore, upregulation of NOX4 in the stressed heart *in vivo* specifically activates NRF2 and its downstream antioxidant signaling cascade, which serves to limit oxidative stress, mitochondrial DNA damage and cardiomyocyte death (17). As recently demonstrated, NRF2 also contributes to the physiological role of NOX4 in the heart as an activator of NRF2 in order to support normal physical exercise (60). Specifically, the increased levels of NOX4 observed following acute exercise result in the concomitant activation of the NRF2 transcription factor and its antioxidant target genes for optimal increments in heart performance during exercise. The pairing between NOX4 and NRF2 triggers an adaptive response to maintain redox state and support mitochondrial and, hence, contractile function in the exercised heart.

**Figure 1:**
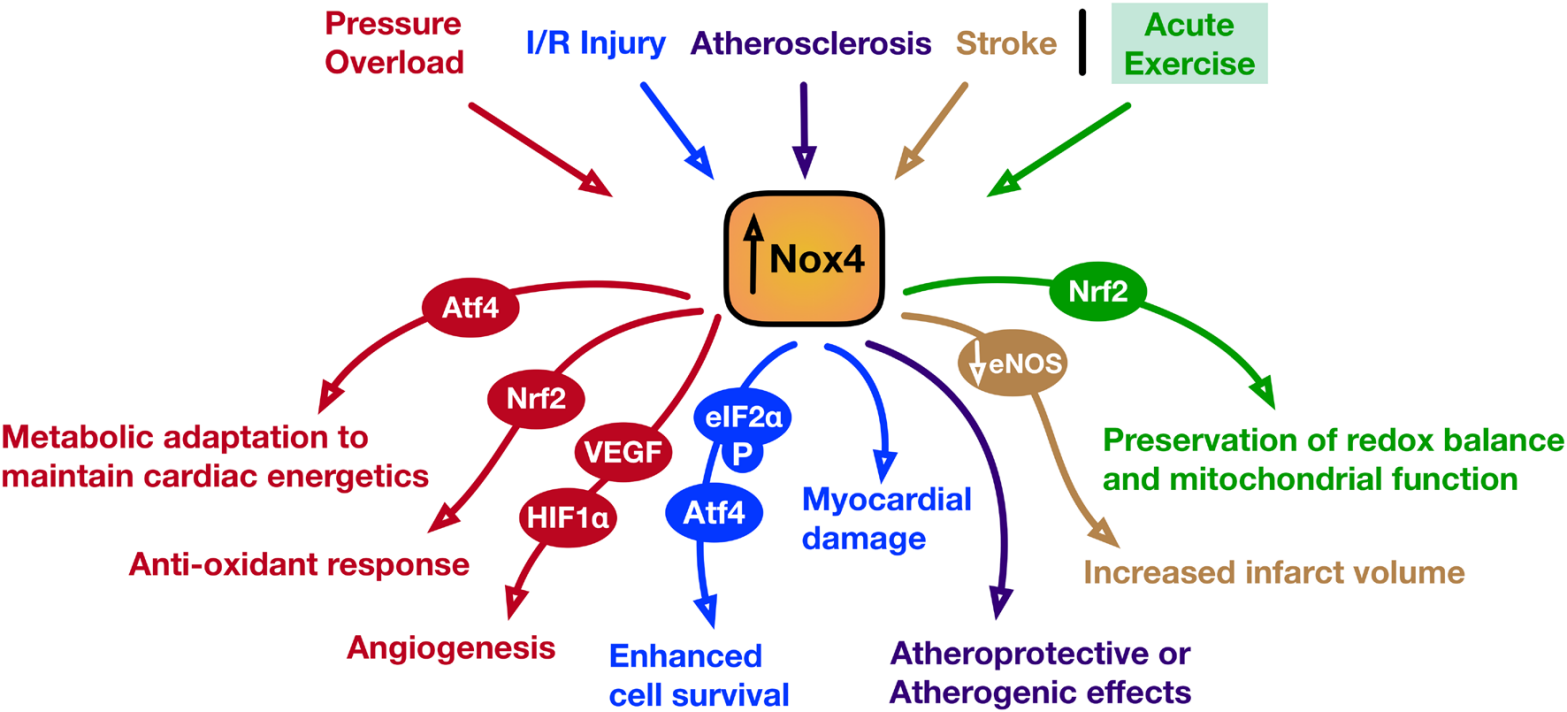
The pathophysiological and physiological effects of NOX4 under various conditions of cardiovascular stress. Summary of the key signaling events that have been identified to be regulated by NOX4 that are engaged downstream of various pathological (pressure overload; red, I/R injury; blue, atherosclerosis; purple, stroke; brown) or physiological (acute exercise; green) cardiovascular stresses.

The cardioprotective effects of NOX4 have also been attributed to regulation of the hypoxia-induced HIF1a. The transcription factor Hif1a and VEGF signaling mediate cardiac remodeling and hypertrophy and promote angiogenesis to protect the stressed heart (61, 62). Indeed, NOX4 is protective against cardiac decompensation during hemodynamic overload via the activation of HIF1a, possibly due to inhibition of prolyl hydroxylases (PHDs) and release of VEGF from cardiomyocytes and/or ECs (15). As a result of the actions of NOX4 myocardial capillary density is preserved in the pressure-overloaded heart.

Finally, studies have demonstrated the interplay between NOX4 and the ATF4 transcription factor in the diseased heart. Autophagy is an essential survival mechanism in the energy-deprived heart. Indeed, activated NOX4 and subsequent generation of ROS promote autophagy in response to energy stress (e.g., fasting) through activation of the PKR-like ER kinase (PERK) pathway by suppression of prolyl hydroxylase 4 (PHD4) (18). Moreover, in the pressure-overloaded heart, hypertrophic remodeling includes a switch in the preferred energy substrate from fatty acids to glucose. The upregulation of NOX4 levels in the overloaded heart reprograms cardiac substrate metabolism in order to maintain cardiac energetics under conditions of stress. Nabeebaccus *et al*. recently reported a NOX4- and ATF4-dependent upregulation of the hexosamine biosynthetic pathway, which enhances fatty acid utilization via the attachment of O-linked N-acetylglucosamine (O-GlcNAcylation) to the fatty acid transporter CD36 (63). This is a novel identification of a NOX4-dependent coordinated reprogramming of cardiac fatty acid and glucose metabolism, demonstrating the optimal compartmentalization of glucose as an adaptive pathway in the hemodynamically overloaded heart.

## Conclusion

The diverse outcomes of NOX4 activation in the cardiovascular system (Fig. 1) are one of the reasons why non-specific, antioxidant approaches have failed to demonstrate any positive outcomes in heart disease. The interplay between redox pools with detrimental and/or beneficial effects exemplifies the requirement for the identification of specific targets for therapeutic manipulation (i.e. activation of NOX4-regulated pathways). Better understanding of the ROS-regulated signaling pathways and data on humans will determine the potential for clinical translation.

## Declaration of interest

The authors declare that there is no conflict of interest that could be perceived as prejudicing the impartiality of this review.

## Funding

This work was supported by the British Heart Foundationhttp://dx.doi.org/10.13039/501100000274 (grant numbers PG/16/30/32129, RG/13/11/30384, and FS/14/77/30913); in part by the Department of Health via a National Institute for Health Research (NIHR) Biomedical Research Centrehttp://dx.doi.org/10.13039/100014461 award to Guy’s & St Thomas’ NHS Foundation Trusthttp://dx.doi.org/10.13039/501100004941 in partnership with King’s College London and King’s College Hospital NHS Foundation Trusthttp://dx.doi.org/10.13039/100010872 and a Fondation Leducqhttp://dx.doi.org/10.13039/501100001674 Transatlantic Network of Excellence.
